# Design and Analysis of a Novel 24 GHz Up-Conversion Mixer with Improved Derivative Super-Position Linearizer Technique for 5G Applications

**DOI:** 10.3390/s21186118

**Published:** 2021-09-12

**Authors:** Abrar Siddique, Tahesin Samira Delwar, Prangyadarsini Behera, Manas Ranjan Biswal, Amir Haider, Jee-Youl Ryu

**Affiliations:** 1 Department of Smart Robot Convergence and Application Engineering, Pukyong National University, Busan 48513, Korea; abrarkhokhar.iiui@gmail.com (A.S.); samira.fset@gmail.com (T.S.D.); prangyadarsini.behera@gmail.com (P.B.); mrbiswal13@gmail.com (M.R.B.); 2 Department of Intelligent Mechatronics Engineering, Sejong University, Seoul 05006, Korea

**Keywords:** 5G, wireless communication, transmitter, up-conversion mixer, Improved Derivative Super-Position

## Abstract

A 24 GHz high linear, high-gain up-conversion mixer is realized for fifth-generation (5G) applications in the 65 nm CMOS process. The mixer’s linearity is increased by applying an Improved Derivative Super-Position (I-DS) technique cascaded between the mixer’s transconductance and switching stage. The high gain and stability of amplifiers in the transconductance stage of the mixer are achieved using novel tunable capacitive cross-coupled common source (TCC-CS) transistors. Using the I-DS, the third-order non-linear coefficient of current is closed to zero, enhancing the linearity. Additionally, a TCC-CS, which is realized by varactors, neutralizes the gate-to-drain parasitic capacitance (C_gd_) of transistors in the transconductance stage of the mixer and contributes to the improvement of the gain and stability of the mixer. The measured 1 dB compression point OP_1_dB of the designed mixer is 4.1 dBm and IP_1_dB is 0.67 dBm at 24 GHz. The conversion gain of 4.1 dB at 24 GHz and 3.2 ± 0.9 dB, from 20 to 30 GHz is achieved in the designed mixer. Furthermore, a noise figure of 3.8 dB is noted at 24 GHz. The power consumption of the mixer is 4.9 mW at 1.2 V, while the chip area of the designed mixer is 0.4 mm${}^{2}$.

## 1. Introduction

**Figure 1:**
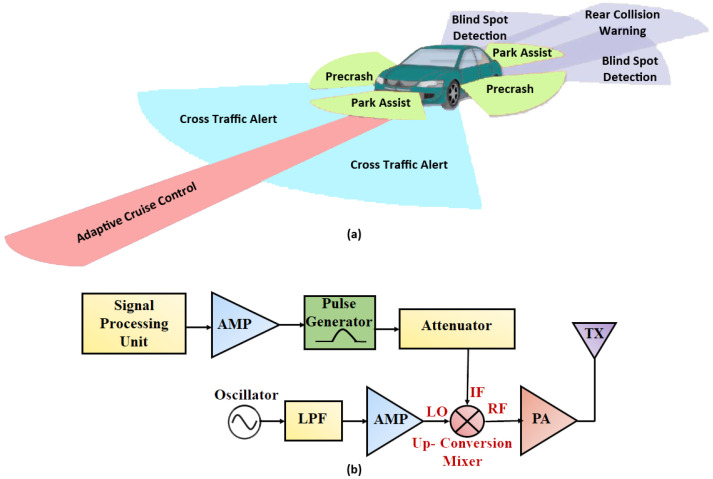
(**a**) Automobile radar application, (**b**) radar transmitter design.

In recent studies, the demand for 5G communication systems was shown to have enormously increased. There are many 5G mobile communication devices available; some of them are wireless broadband internet, cellular phones, etc. Low power, low voltage and highly linear RF circuits attract considerable attention in regard to prolonging the battery life of communication systems. The 5G system is becoming a necessity in wireless communication [1,2]. Existing technologies, including LTE and 4G mobile communications, cannot satisfy the increasing demands for a fast data rate, low latency and larger capacity. The 5G system is 1000 times faster than 4G technology. Furthermore, 5G accumulates high data traffic capacity. Many advanced communication applications require a high capacity, high data rate communication system, and the 5G technologies fulfill these requirements. A frequency band from 20 GHz to 30 GHz has been relevant in 5G radar applications [3]. Vehicular Radar systems can give useful insight into the other millimeter-wave applications. Additionally, in the automotive sector, radars are widely employed for the development of cars, which offer the basis for a secure and intelligent transport system [4]. The 5G frequency spectrum gained a great deal of attention because of its prospective usage of automobile vehicles for radar applications [5]. Nowadays, automotive radar is regarded as one of 5G technology’s most important vertical markets. Thus, in [6], the author also describes a 5G transceiver radar application (24 GHz) that achieves a high data rate and wide bandwidth. The radar applications in automobile vehicles and radar transmitter design are shown in Figure 1a,b, respectively.

**Figure 2:**
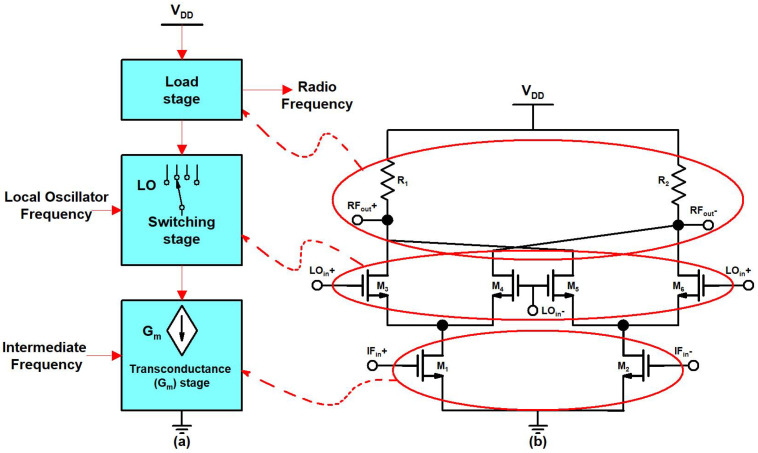
(**a**) Block diagram, (**b**) schematic of the conventional of mixer.

High integration, low power, low noise, and high linearity are the main design requirements in the 5G transmitter design. An up-conversion mixer is the main circuit block in a 5G radar transmitter design. Designing a highly linear, high-gain up-conversion mixer is a fundamental challenge in transmitter design. A highly linear, high-gain up-conversion mixer is compulsory to overcome the transmitter linearity limitation. Linearity is an essential characteristic of an up-conversion mixer because a transmitter with a mixer with low linear output requires a buffer amplifier to drive a power amplifier that degrades the linearity. So, a linear up-conversion mixer with a high-output 1 dB compression point (OP_1_dB) benefits the transmitter design. The block diagram and schematic of the conventional mixer are shown in Figure 2a,b, respectively.

### Related Literature Survey

In general, mixers are often categorized as passive and active mixers. Passive mixers can be used to generate an intermediate frequency (IF) for a local oscillator (LO) driving signal by using passive switches. Passive mixers could attenuate the signal. On the other hand, active mixers offer a positive conversion gain (CG), while passive mixers do not [7]. However, passive mixers are simple. They have zero power consumption, are highly linear, have good NF, but they require a high LO power. Active mixers offer excellent CGs, good port isolation, and minimum NF and LO power, unlike passive mixers. When analyzing all these facts, active mixers are preferred over passive mixers.

Recently, many active CMOS up/down mixers [8] were reported with different topologies that enhanced the linearity and gain performances. Zumbahlen et al. [9] proposed a circuit with a minimum amount of noise and strong linearity, while Siddiqi et al. [10] described the mixer design as having a minimal NF but inadequate CG and linearity. Additionally, in [11], the proposed mixers have minimal power consumption and high linearity; however, these mixers are often employed at the expense of port isolation, and the shortcoming is that they require a high LO power. In [12], the author designed the most popular Gilbert mixer, showing high isolation. Another previous study [13] described a mixer with high performance in terms of CG and NF but at the expense of linearity. In [14,15], the LO and radio frequency (RF) signals were applied at the drain and gate terminals of the transistor, but these mixers suffered with poor linearity. In [16,17], to enhance the linearity, a CMOS mixer operating at 2.4 GHz with a derivative superposition (DS) technique and a mixer design with input active balun is presented. However, this mixer design increases the power consumption. In [18], a 60 GHz mixer with direct up-conversion architecture achieved a gain of 4.5 dB; however, it had poor linearity and power dissipation of 15.1 mW. Various other up-conversion 24 GHz CMOS mixers are described in [19,20] and showed a degraded linearity performance.

In [21], an up-conversion mixer was designed in a 90 nm CMOS process with dual pMOS and nMOS cross-coupled transistors to gain of 2.1 dB due to the current injection and negative resistance provided by these transistor pairs, but the linearity performance was degraded and it exhibited the 1 dB compression point of −10 dBm. Further, a Tanh-mixer with N = 3 is reported in [22], achieving a high gain of 3.8 dB. This mixer achieved an excellent gain result but the isolation and power consumption of 21.1 dB and 107 mW, respectively, degraded the overall performance mixer circuit.

For high gain and excellent isolation, the Gilbert-cell mixer circuit is most commonly used [23,24,25,26,27]. The linearity of the Gilbert-cell mixer depends on the input transconductance stage, which consists of a transconductance amplifier (TA). In [25], a mixer design with cross-coupled voltage bias and offset TA is presented, and its transconductance depended on the linearly varied bias offset voltages. However, this technique does not adhere with the technology scaling, which required low supply voltages. In [22,27], a mixer design was fabricated with the implementation of a transconductance stage based on a negative-feedback loop to address the linearity, but the negative-feedback loop decreased the gain of the circuit.

Furthermore, to achieve high linearity and gain, many CMOS mixers are designed by using different linearity techniques such as a dual transconductance (G_m_) boosting path along with dual n/PMOS switches [28], a cascode folded mixer [29], the insertion of source-degenerated resistors [30], a class-AB amplifier G_m_ stage [31,32,33], employing a diode linearity technique [34] and integrating high-order harmonic termination [35]. All these linearity techniques either make the mixer circuit complex or lower the gain.

**Figure 3:**
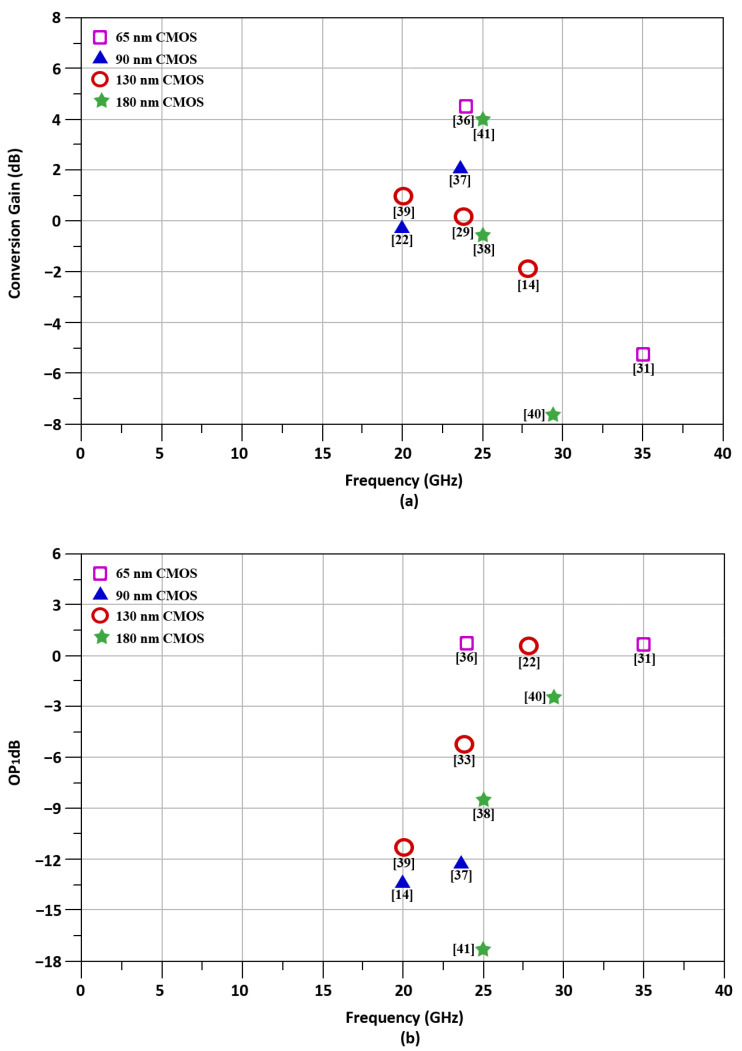
Literature survey of up-conversion mixers [14,22,31,33,36,37,38,39,40,41] with operating frequencies of 15–35 GHz. (**a**) Conversion gain, (**b**) OP_1_dB.

According to the author’s best knowledge, literature based on the design of CMOS up-conversion mixers operating at a particular 24 GHz frequency with high linearity and high gains is not frequently published. Figure 3 describes the literature survey of up-conversion mixers in terms of conversion gain, and OP_1_dB operating within the frequency range of 15–35 GHz. As our paper deals with the specific 24 GHz frequency, we made a fair comparison between the other published articles in a similar frequency range for the simplicity of the paper. Hence, the prior research shows that while the frequency range is from 15 to 35 GHz, all the authors work on the one specific parameter, i.e., to increase the conversion gain or to enhance the linearity of the up-conversion mixers. Only the previous research in [21] presents a 24 GHz up-conversion mixer that achieved a high conversion gain and high linearity concurrently. This paper’s limitation is that it is confined to this idea, and it does not discuss technical details thoroughly. Furthermore, the application is limited to automotive radar applications, while our work deals with 5G applications at 24 GHz frequencies.

In our work, the novelty lies in the fact that we are able to achieve high gain and high linearity simultaneously, with the help of our newly proposed technique. Hence, the designed up-conversion mixer includes tunable capacitive cross-coupled common source (TCC-CS) transistors to increase the gain. Furthermore, The I-DS technique also boosts the linearity. By using TCC-CS and I-DS techniques, the designed mixer archives high gain and high linearity with moderate power consumption. The up-conversion mixer achieves a peak CG of 4.1 dB at 24 GHz and the measured IP_1_dB of the designed mixer is 0.67 dBm at frequencies from 20 to 30 GHz, which is one of the highest-frequency up-conversion mixers among 65 nm technologies reported for 5G applications.

The remaining part of the paper is set out as follows. Section 2 describes the proposed up-conversion mixer system design. The results and discussion are shown in Section 3. The conclusion is finally drawn in Section 4.

## 2. Proposed Up-Conversion Mixer Design

**Figure 4:**
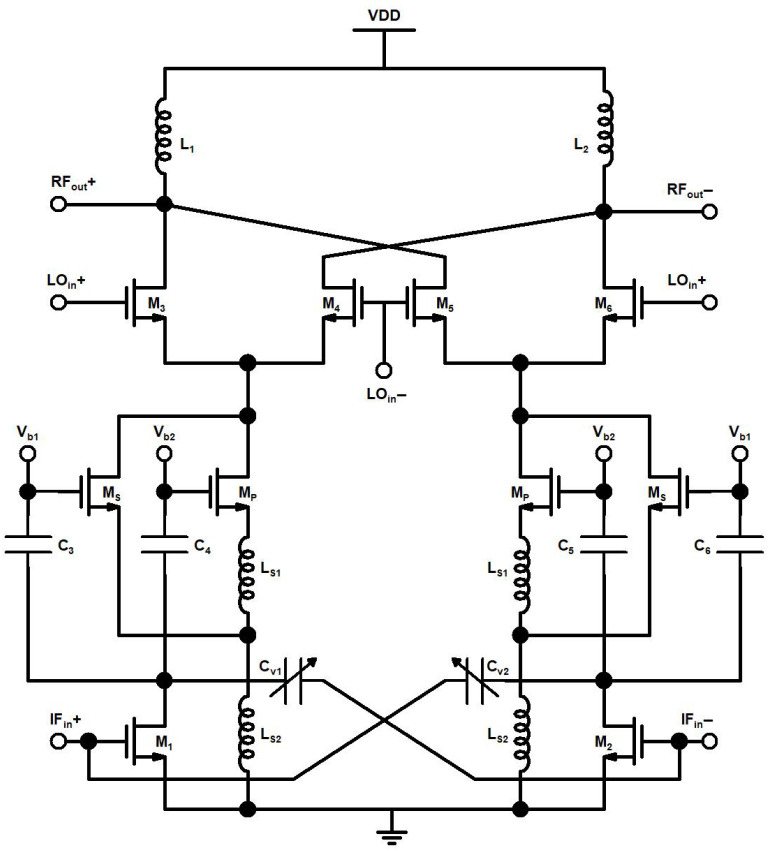
Proposed up-conversion mixer.

The proposed up-conversion mixer’s schematic is shown in Figure 4. The 2.4 GHz IF input signal is amplified in the G_m_ stage, which consists of TCC-CS and I-DS. The TCC-CS is implemented with CS transistors M_1_, M_2_, and, varactors C_v1_, C_v2_, the varactors are biased with tunning voltage V_t_. The I-DS contains primary transistors, M_P_, secondary transistors, M_S_, dc blocking capacitors, C_3_–C_6_ and source-degenerated inductors, L_s1_ and L_s2_. The L_s1_, and L_s2_ are source-degenerated inductors of the primary and secondary transistors of I-DS. The linearized and the amplified output signal of the G_m_ stage is fed into the switching stage of the mixer, which comprises transistors M_3_–M_6_, and an LO signal of 21.6 GHz is applied at the gate terminal of these transistors. The G_m_ stage signal is translated to a 24 GHz RF output signal at the switching stage. At the RF output stage, the RF output buffer (not shown in the mixer schematic (Figure 4) for simplicity) is designed in a push/pull configuration by using a PMOS transistor (M_pb_), NMOS transistor (M_nb_) and feedback resistor (R_f_) to match the 50 Ohm resistance.

**Figure 5:**
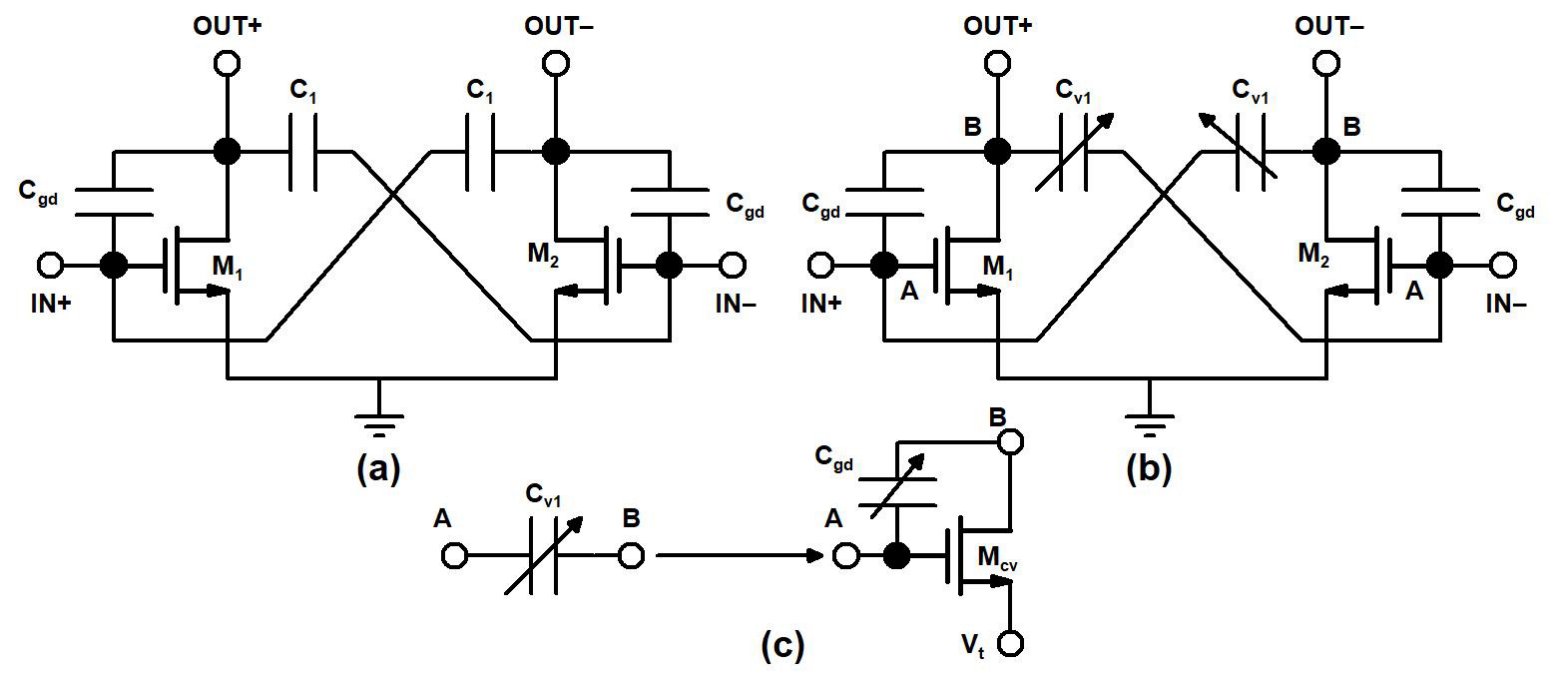
(**a**) Conventional capacitive cross-coupling neutralization, (**b**) TCC-CS and (**c**) varactor realization in TCC-CS.

In the designed mixer, TCC-CS topology provides a high gain and stability, downgraded in CS topology due to parasitic gate to drain capacitances (C_gd_) of transistor M_1_ and M_2_. The conventional capacitive neutralization proposed TCC-CS and the realization of a varactor in TCC-CS is shown in Figure 5a–c, respectively.

A cross-coupled capacitor C_v1_ connected between the drain terminal of M_1_ and the gate terminal, M_2_m acts as a negative equivalent to capacitor C_v2_, which is connected between the drain terminal of M_2_ and gate terminal, M_1_. The capacitance, C_v1_ and C_v2_, is used to nullify the parasitic capacitance, C_gd1_, of M_1_ and C_gd2_ of M_2_ as the signals across the varactor and C_gd_ are opposite in phase. The tunning voltage, V_t_, of varactors applied at the source of transistor M_cv_ (Figure 5c) to cancel the effect of C_gd_ is small so that transistor operates in the subthreshold region and does not contribute much to the overall power consumption of mixer.

**Figure 6:**
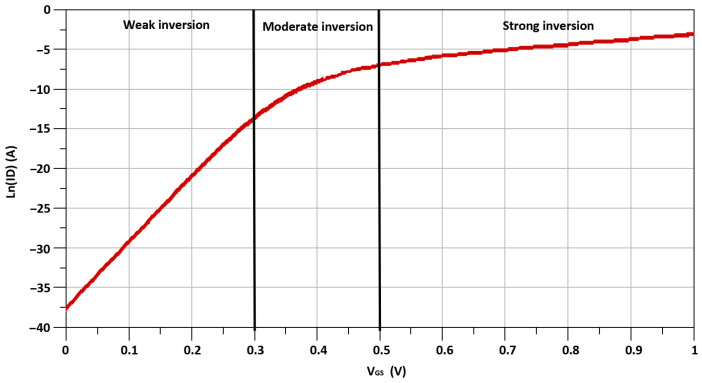
I–V DC characteristic curve of the transistors with drain current Ln(ID) on the vertical-axis.

**Figure 7:**
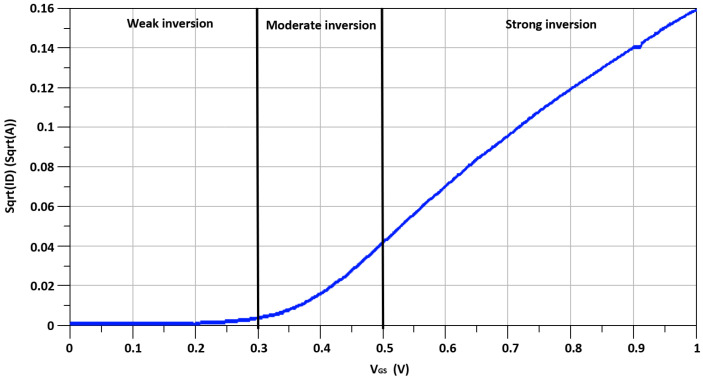
I–V DC characteristic curve of the transistors with drain current sqrt(ID) on the vertical-axis.

While the inclusion of I-DS in between TCC-CS topology and switching stage enhances the proposed mixer’s linearity, in I-DS, secondary transistor, M_s_, is connected parallel to the primary transistor, M_P_. The secondary transistor of I-DS, M_s_, operates in the moderate-inversion region with biasing voltage, V_b1_, instead of the conventional DS technique where secondary transistors operate in the weak-inversion region. Meanwhile, primary transistors, M_P_, operates in the strong-inversion region with biasing voltage, V_b2_. The moderate inversion region biasing of M_s_ helps to reduce the gate noise, which is inversely proportional to the current Ids_3_ of M_s_. The I–V DC characteristic curve of the MOS transistors of 65 nm CMOS technology is shown in Figure 6 and Figure 7. As illustrated by the I–V DC characteristic curve, the transistor operating region can be categorized into three regions: moderate-inversion, strong-inversion and weak-inversion, depending on the transistor’s biasing conditions.

**Figure 8:**
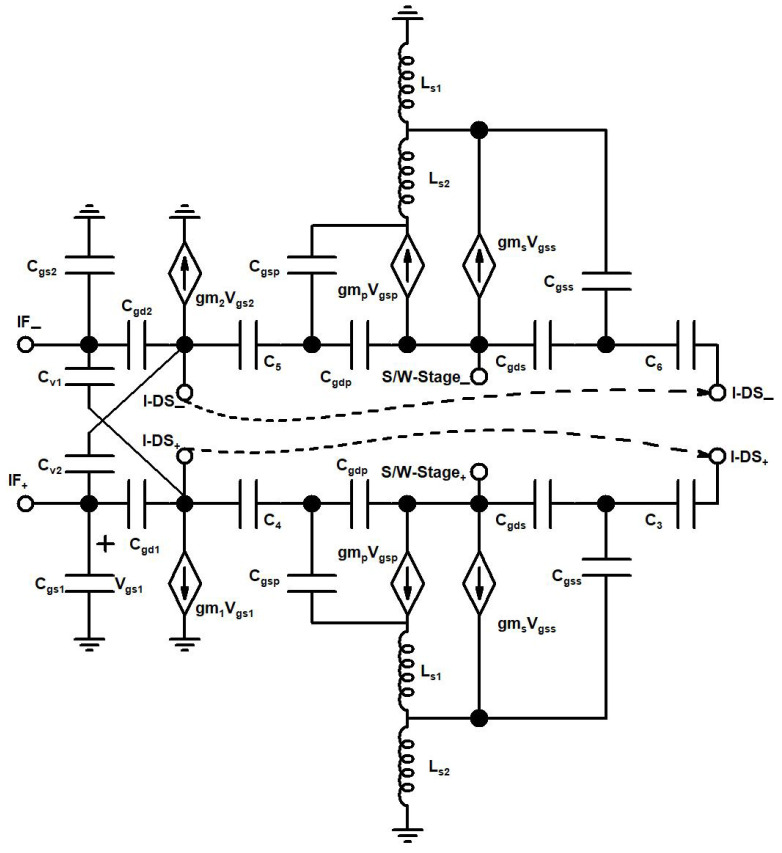
Small signal model of the transconductance stage.

The small signal model of the transconductance stage of the designed mixer is shown in Figure 8.

**Figure 9:**
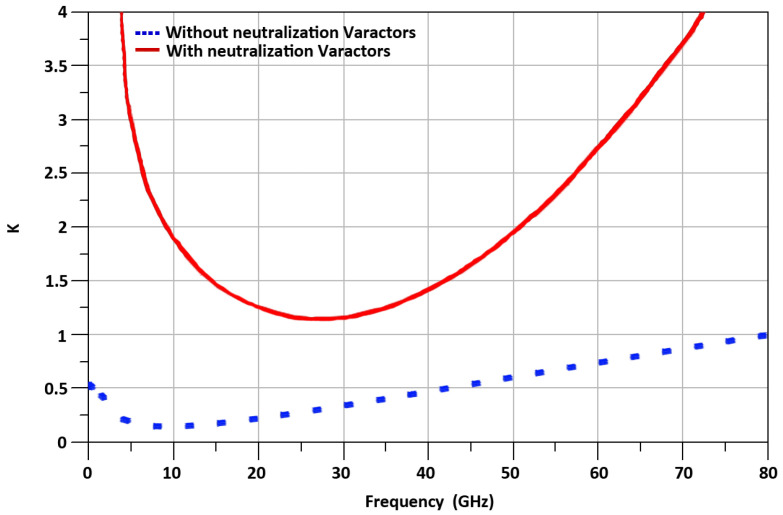
Stability Factor K.

The stability factor, *K*, of M_1_ transistor of TCC-CS is shown in Equation (1) and the simulated K is shown in Figure 9. The amplifier is stable if *K* > 1 [14].

(1) $$K=\frac{2+\omega^{2}{\left(C_{gd1}-C_{v1}\right)}^{2}}{\omega \left|C_{gd1}-C_{v1}\right|\sqrt{\omega^{2}{\left(C_{gd1}-C_{v1}\right)}^{2}+g_{m1}^{2}}}$$

**Figure 10:**
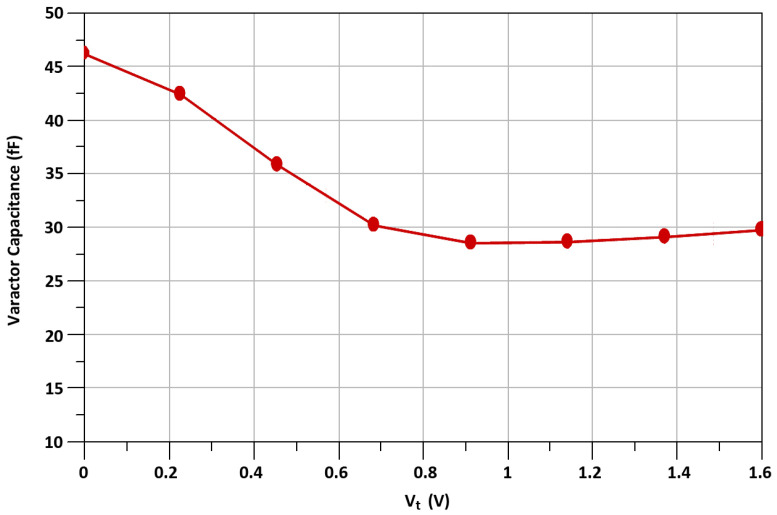
Varactor capacitance versus voltage V_t_.

**Figure 11:**
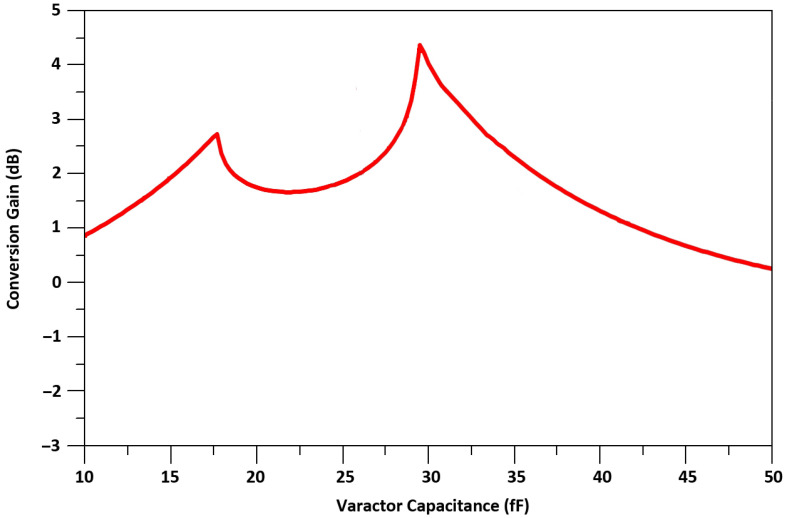
Theoretical result of G.

Equation (1) shows that when varactor’s capacitance C_v1_ is equal to C_gd1_, the stability factor is maximum. As we use a varactor’s capacitance, to neutralize the C_gd_, which can be controlled externally, the PVT variations do not affect the stability of M_1_. The simulated varactor capacitance versus the voltage, V_t_, is shown in Figure 10. The gain, G, of TCC-CS of the transconductance stage is expressed in Equation (2) and the calculated result of G versus the varator capacitance is shown in Figure 11.

(2) $$\begin{array}{c} G=\left(\frac{\sqrt{\omega^{2}{\left(C_{gd1}-C_{v1}\right)}^{2}+g_{m1}^{2}}}{\left|C_{gd1}-C_{v1}\right|}\right)\left(\frac{2+\omega^{2}{\left(C_{gd1}-C_{v1}\right)}^{2}}{\omega \left|C_{gd1}-C_{v1}\right|\sqrt{\omega^{2}{\left(C_{gd1}-C_{v1}\right)}^{2}+g_{m1}^{2}}}\right)\\ -\sqrt{{\left(\frac{2+\omega^{2}{\left(C_{gd1}-C_{v1}\right)}^{2}}{\omega \left|C_{gd1}-C_{v1}\right|\sqrt{\omega^{2}{\left(C_{gd1}-C_{v1}\right)}^{2}+g_{m1}^{2}}}\right)}^{2}-1} \end{array}$$

Equation (2) shows that the TCC-CS along with stabilization also enhances the gain of the designed mixer. The device sizes of the designed mixer are shown in Table 1.

### 2.1. Linearity Analysis

**Figure 12:**
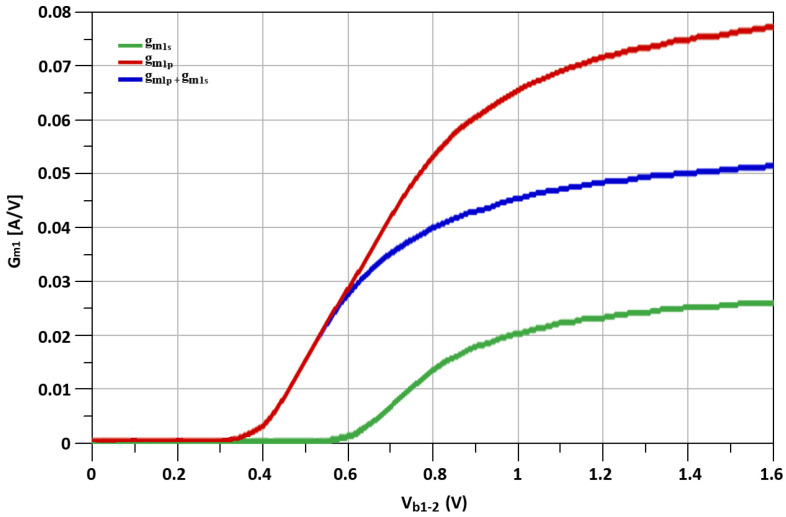
The simulated g_m1s_, g_m1p_ and g_m1s_ + g_m1p_ of M_p_ and M_s_ transistors.

**Figure 13:**
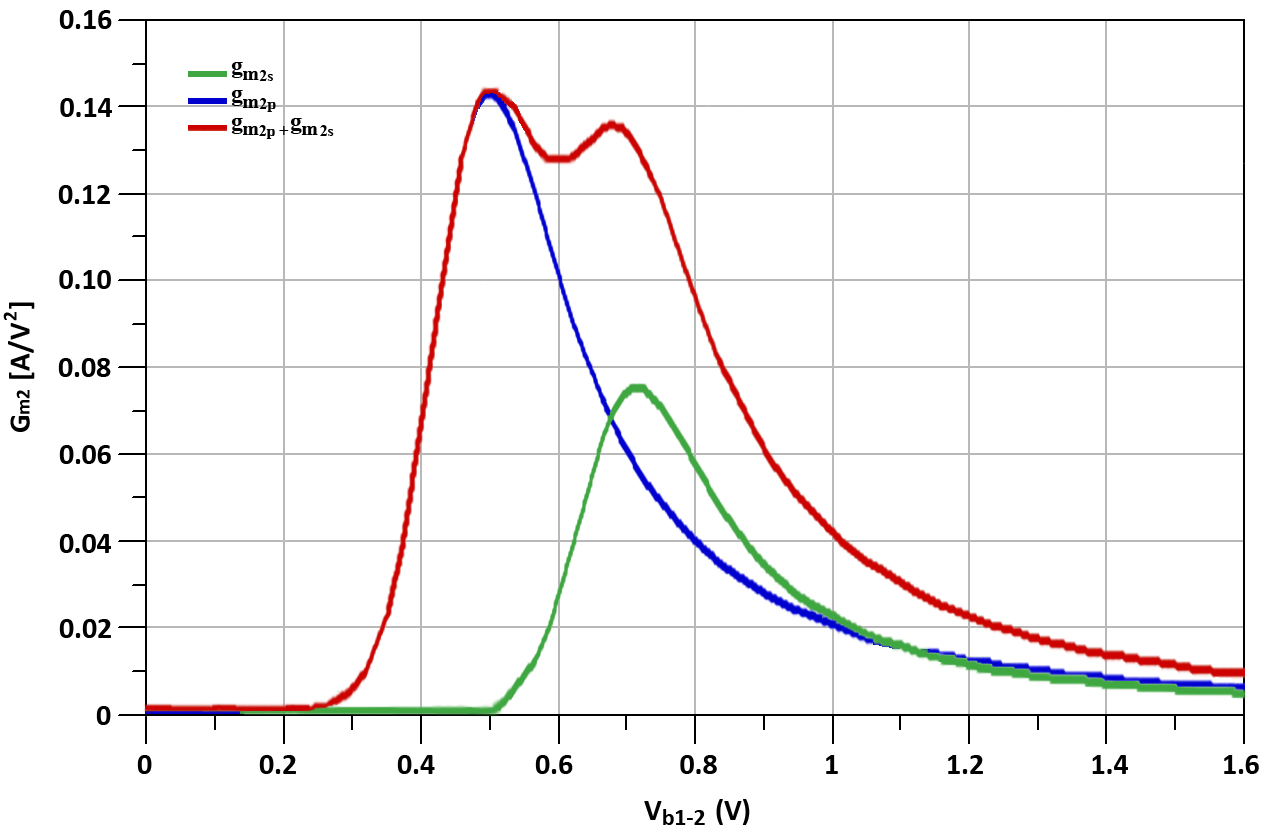
The simulated g_m2s_, g_m2p_ and g_m2s_ + g_m2p_ of M_p_ and M_s_ transistors.

**Figure 14:**
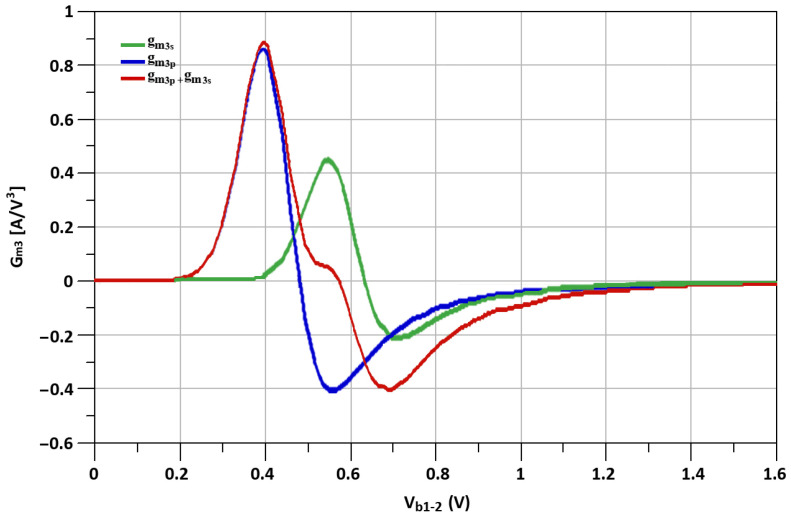
The simulated g_m3s_, g_m3p_ and g_m3s_ + g_m3p_ of M_p_ and M_s_ transistors.

The simulated fundamental transconductances (g_m_), represented as, “g_m1s_”, “g_m1p_” and “g_m1s_ + g_m1p_” of transistors M_s_, M_p_, the second-order transconductance (g_m_’) mentioned as “g_m2s_”, “g_m2p_” and “g_m2s_ + g_m2p_” of transistosr M_s_, M_p_, and the third-order transconductance (g_m_”) noted as, “g_m3s_”, “g_m3p_” and “g_m3s_ + g_m3p_” of transistors M_s_, M_p_ with respect to biasing voltage, V_b_, are shown in Figure 12, Figure 13 and Figure 14, respectively.

When it works in the saturation region, the operating region of the CMOS transistor is classified into three different regions: weak/moderate/strong inversion regions. The primary reason for non-linearity in the CMOS transistor is the transconductance (g_m_) [30]. In the weak-inversion region, the operational speed of the CMOS transistor is slow, but the ratio of g_m_ and drain current (I_d_) is high, while it is reversed in the case of the strong-inversion region of the CMOS transistor. The moderate-inversion region exhibits good operational speed and a good g_m_/I_d_ ratio. The drain to source current (I_ds_) of a CS transistor is shown in Equation (3) [31].

(3) $$I_{ds}=I_{dc}+g_{m1}V_{gs}+g_{m2}V_{gs}^{2}+g_{m3}V_{gs}^{3}+...$$

The g_m2_ and g_m3_ are the primary factors on which the IIP3 of the CMOS transistor depends. The IIP3 for the I-DS technique is shown in Equation (4).

(4) $$IIP3=\frac{2g_{m1s}^{2}\omega^{2}\left[L_{s1}\left(C_{gsp}+C_{gss}\right)+L_{2}C_{gss}\right]}{3\left|\alpha \right|}$$

(5) $$\begin{array}{c} \alpha =g_{m3p}\left(1+j\omega L_{s2}g_{m1s}\right){\left|\left(1+j\omega L_{s2}g_{m1s}\right)\right|}^{2}\left[1+\frac{L_{s2}C_{gss}}{L_{s1}\left(C_{gsp}+C_{gss}\right)+L_{s2}C_{gss}}\right]\\ +g_{m3s}-\frac{2g_{m2s}^{2}}{3g_{m1s}}\left(\frac{1}{1+\left(1/j2\omega \left(L_{s1}+L_{s2}\right)g_{m1s}\right)}\right) \end{array}$$

where C_gss_ and C_gsp_ represent the parasitic gate-source capacitances of M_s_, M_p_ transistors of I-DS. It is shown from Equation (4) that, by selecting proper values for L_s1_ and L_s2_, the effects of g_m2_ on the IIP3 can be reduced, which helps to improve the linearity of the designed mixer.

By carefully determining the sizes of transistors, source-degenerated inductors of the I-DS technique and with proper biasing conditions, the linearity of the proposed mixer is improved. L_s1_ and L_s2_ tune out the second-order non-linear components. At the same time, the third-order non-linear components can be diminished by choosing proper I-DS transistor sizes and biasing conditions [42].

### 2.2. Layout Issues

**Figure 15:**
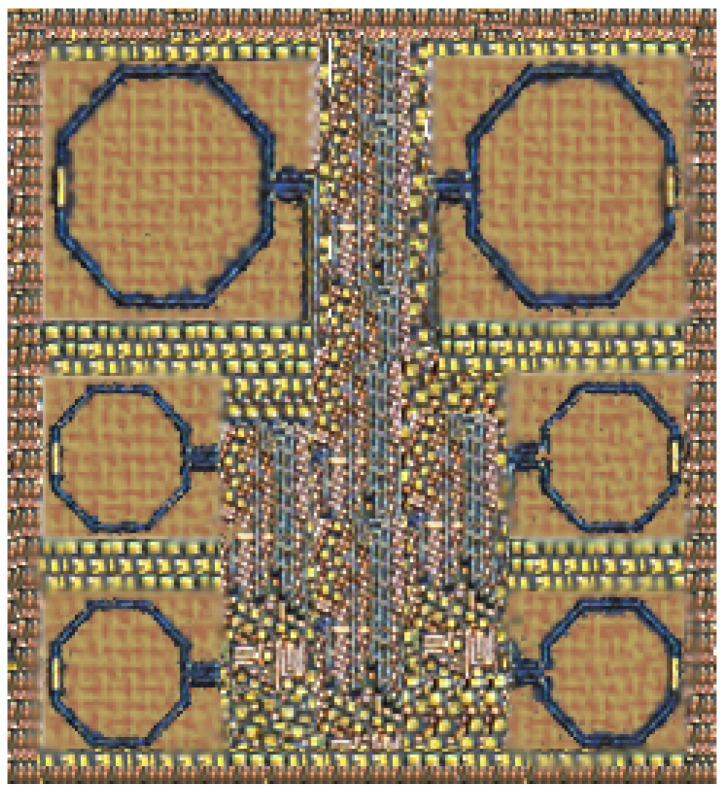
Microphotography of the up-conversion mixer.

The proposed mixer, which comprises the TCC-CS, I-DS technique, is fabricated in 65 nm CMOS technology. The mixer chip microphotograph is shown in Figure 15. The mixer’s chip size is 0.4 mm${}^{2}$ (0.71 × 0.57 mm${}^{2}$), with the exclusion of chip pads. Chip layout is carried out to ensure the stable permanence of the mixer and to reduce the parasitic resistive, capacitive and inductive effects of interconnecting lines and the parasitic capacitive effects of multiple diffusion strips. The size of transistors is divided into multiple fingers to decrease the series resistance and parasitic capacitances of the gate of transistors. Furthermore, due to multiple finger transistors, the nonlinearity due to shunt capacitance decreases and high gain is achieved. The proposed mixer’s ground layer is designed with a multi-layer technique by using different metal layers to develop a low inductive and resistive ground path. Thick and large power lines are designed to achieve the good analog current (AC) coupling between the ground and also to prevent a voltage drop. The metal insulator metal (MIM) with a capacitance of 2.2 fF/μm${}^{2}$ is used to design a capacitor. Meanwhile, inductors of the mixer with a quality factor (Q) equal to 12 are designed by using metal layer 5 of 65 nm CMOS technology with 12 μm thickness. To mitigate the electromagnetic interference (EMI) in between the inductors, a 16 μm ground plane shield from the inductor coil is implemented.

## 3. Results and Discussion

**Figure 16:**
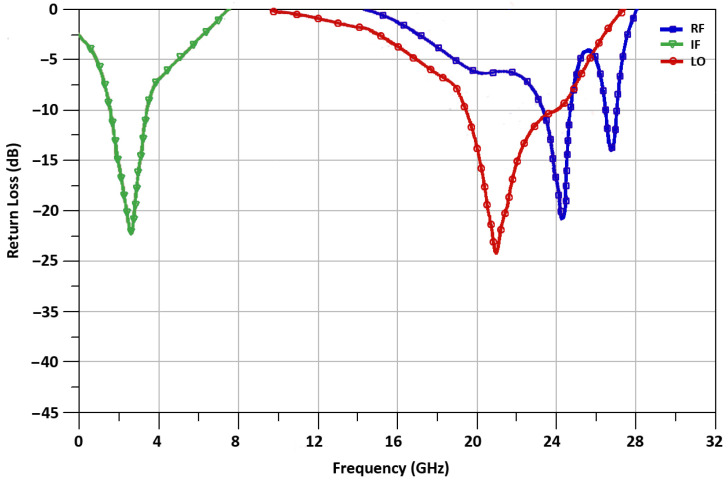
Measured return loss.

The up-conversion mixer is fabricated in 65 nm CMOS technology and the operating characteristics of the mixer are simulated and measured. The measuring probes with a ground–signal–ground–signal–ground (GSGSG) pattern are used to measure the characteristics of the mixer. The mixer operates at 1.2 V dc voltage supply, while it consumes power equal to 4.9 mW. The measured return loss of the proposed mixer is depicted in Figure 16. The IF port of the mixer at 2.4 GHz shows a return loss of −22.6 dB, the RF port of the mixer at 24 GHz shows a return loss of −20.7 dB and the LO-port of the mixer at 21.6 GHz shows a return loss of −24.8 dB.

**Figure 17:**
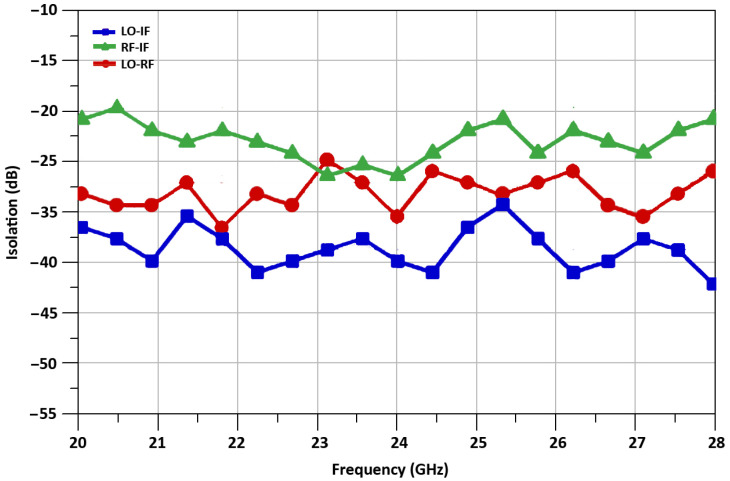
Isolations between mixer’s ports.

The isolation between LO-port to RF-port, RF-port to IF-port, and LO-port to IF-port of the mixer at 24 GHz is −35.1 dB, −27.3 dB, −40 dB, respectively, and it is shown in Figure 17.

**Figure 18:**
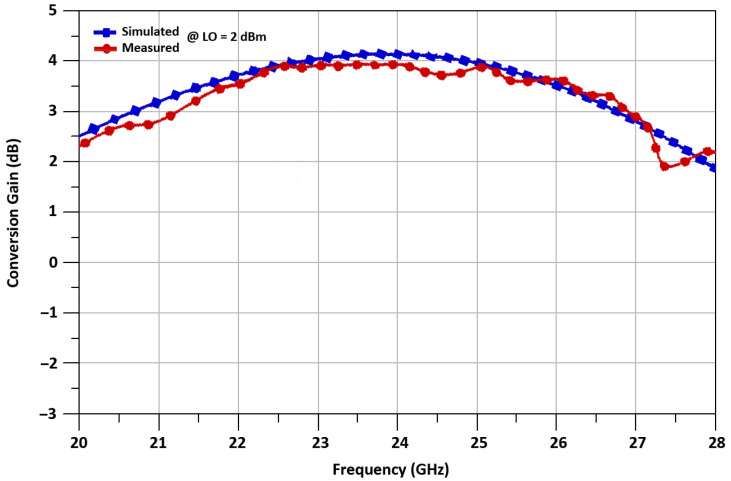
Conversion gain vs. frequency of the mixer.

**Figure 19:**
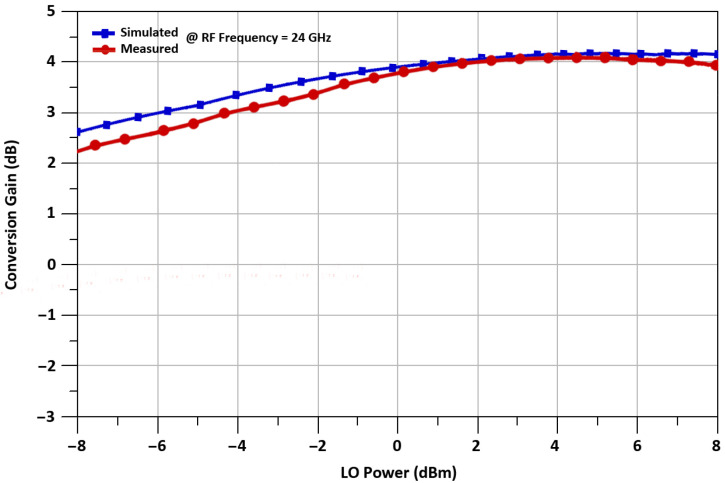
Conversion gain vs. LO power of the mixer.

The proposed mixer’s conversion gain is simulated and measured for 24 GHz up-converted RF frequency, with the 21.6 GHz LO frequency, and 2.4 GHz IF frequency. The conversion gain increases with the increment of the LO power, but for low power operation, LO is selected to be 2 dBm. The maximum measured conversion gain at 24 GHz RF frequency is 4.1 dB. The conversion gain of a mixer is equal to 3.2 ± 0.9 dB, versus frequency from 20 to 30 GHz is illustrated in Figure 18, while conversion gain versus LO power from −8 dBm to 8 dBm is shown in Figure 19.

**Figure 20:**
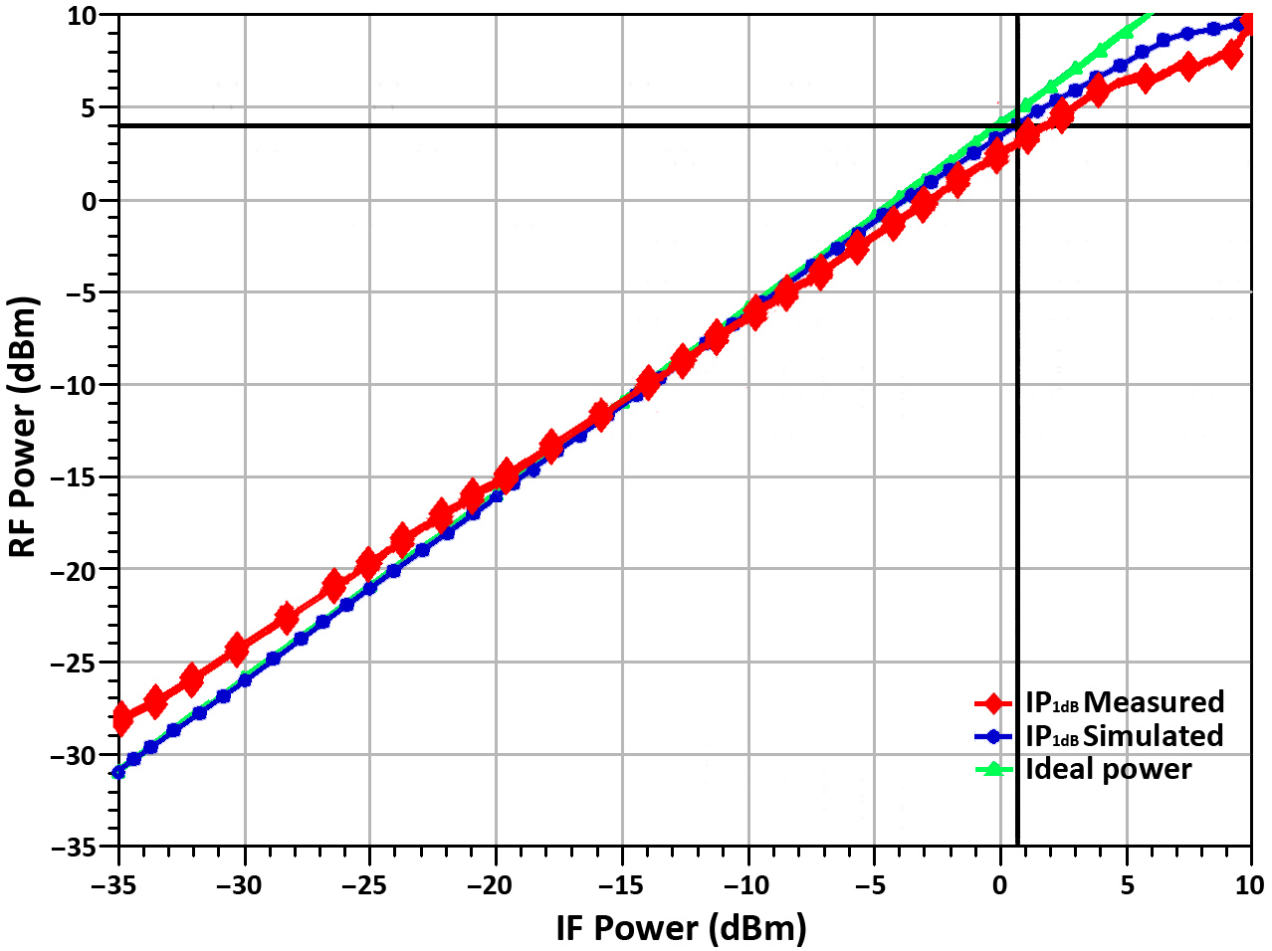
RF output power vs. IF input power.

The linearity result of the proposed up-conversion mixer is illustrated in Figure 20, which shows input power versus output power curves. The mixer shows a measured OP_1_dB of 4.1 dBm, and the IP_1_dB is 0.67 dBm, respectively, demonstrating good linear performance.

**Figure 21:**
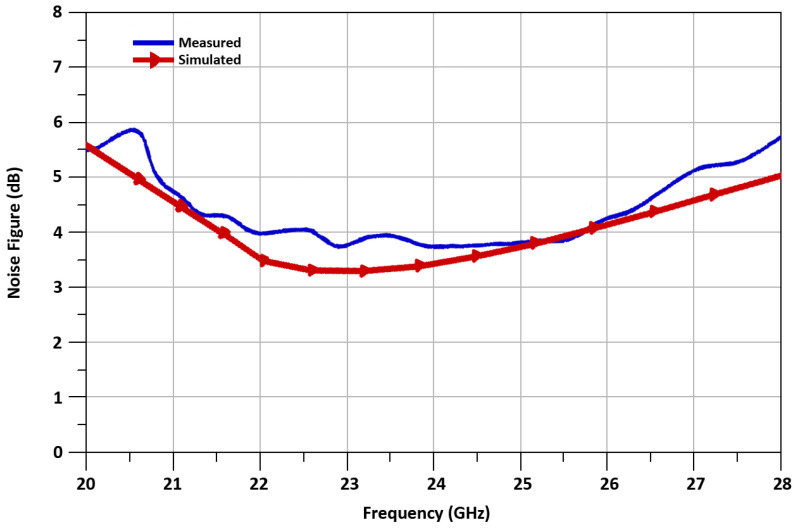
Measured noise figure versus RF frequency.

**Figure 22:**
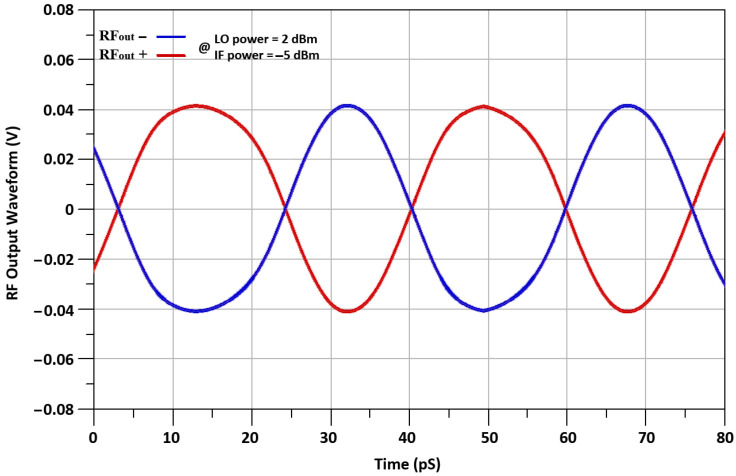
Output voltage waveform.

The noise figure (NF) performance of the mixer is indicated in Figure 21. The NF of 3.8 dB at RF frequency of 24 GHz is achieved. The NF is comparatively high because of the insertion of extra transistors of I-DS transistors. The up-converted RF_out+_ and RF_out-_ transient wave-forms of the designed mixer are shown in Figure 22, where up-converted RF output signal is 24 GHz. The peak–peak voltage swing of RF signal of the mixer is equal to 80 mV.

### Proposed Mixer vs. State-of-the-Art Designs

The result summary of the mixer is listed in Table 2 and compared to already-published state-of-the-art designs of CMOS mixers.

## 4. Conclusions

A 24 GHz up-conversion mixer using 65 nm CMOS technology is proposed for 5G automobile radar applications. This paper aimed to simultaneously increase mixer gain and linearity in the 24 GHz frequency range. Therefore, we proposed a new tunable capacitive cross-coupled common source technique and linearizing I-DS technique. By using TCC-CS in the transconductance stage of the mixer, the gain and stability of the mixer were improved. Furthermore, the I-DS mitigates the third-order nonlinear coefficient and enhances linearity. The measured OP_1_dB of the designed mixer is 4.1 dBm, with a conversion gain of 4.1 dB at 24 GHz and 3.2 ± 0.9 dB, at frequencies from 20 to 30 GHz, and a noise figure of 3.8 dB at 24 GHz. The mixer only consumes 4.9 mW at 1.2 V. We believe that the proposed mixer has high linearity, high gain, and low DC power consumption at 24 GHz, and is best suitable for low-power 5G automobile radar applications.

## Figures and Tables

**Table 1:** Designed mixer circuit component values.

Element	Dimension
M_1_, M_2_	31 μm/65 nm
M_p_	53 μm/65 nm
M_s_	41 μm/65 nm
M_3_–M_6_	37 μm/65 nm
M_cv_	34 μm/65 nm
L_1_–L_2_	175 pH
L_s1_	70 pH
L_s2_	90 pH
C_3_–C_6_	45 fF
C_v1_–C_v2_	29.3 fF @ V_t_ = 0.75 V

**Table 2:** Comparison summary for recently reported results.

Ref.	Process (nm)	RF Freq. (GHz)	Gain (dB)	OP_1_dB	Chip Area (mm${}^{2}$)	Power Consumption (mW)	NF
[19], 2015	130	23.4–29.2	−1.9	0.3	0.8	38	NA
[21], 2021	65	24	4.7	0.41	0.42	5.2	3.8
[43], 2017	65	27.5–43.5	−5	0.42	0.686	14	NA
[44], 2008	65	60	−6.5	−5	0.98	29	NA
[45], 2006	130	18–28	0.7	−5.2	0.46	22.8	NA
[46], 2019	65	17–43	1.6	NA	0.5	NA	12.4
[47], 2006	180	3.1–10.6	10	NA	1	NA	10
[48], 2012	180	2.4	7.1	NA	0.4	4.5	11.9
[49], 2007	250	3.5	3.8	NA	0.34	3.5	8
[This Work]	65	24	4.1	4.1	0.4	4.9	3.8

## Data Availability

Not applicable.

## References

[1] Qayyum J.A., Albrecht J.D., Ulusoy A.C. (2019). A compact V-band upconversion mixer with −1.4 dBm OP1dB in SiGe HBT technology. IEEE Microw. Wirel. Components Lett..

[2] Ayesha A., Rahman M., Haider A., Majeed Chaudhry S. (2021). On Self-Interference Cancellation and Non-Idealities Suppression in Full-Duplex Radio Transceivers. Mathematics.

[3] Siddique A., Delwar T.S., Kurbanov M., Ryu J.Y. (2020). Low-power low-phase noise VCO for 24 GHz applications. Microelectron. J..

[4] Chen Q.B., Schaeffer J.K. 22FDX Embracing IoT, 5G, and Automotive Applications-A Perspective through Global Research. Proceedings of the 2019 IEEE SOI-3D-Subthreshold Microelectronics Technology Unified Conference (S3S).

[5] Siddique A., Ryu J.Y. (2020). A 24 GHz frequency synthesizer for automotive collision avoidance radar. Int. J. Electron. Lett..

[6] Li D., Xia Q., Huang J., Li J., Chang H., Sun B., Liu H. (2021). A 24 GHz Direct Conversion Receiver for FMCW Ranging Radar Based on Low Flicker Noise Mixer. Electronics.

[7] Voltti M., Koivisto T., Tiiliharju E. Comparison of active and passive mixers. Proceedings of the 2007 18th European Conference on Circuit Theory and Design.

[8] El-Desouki M.M., Qasim S.M., BenSaleh M.S., Deen M.J. (2015). Toward realization of 2.4 GHz balunless narrowband receiver front-end for short range wireless applications. Sensors.

[9] Zumbahlen H. (2008). Linear Circuit Design Handbook.

[10] Siddiqi A.A. (2000). Design Methodology and Investigation of GHz Range CMOS RF Mixers. Master’s Thesis.

[11] Asad B.Z.J. (2014). Low-Noise 24 GHz 0.15 μm GaAs pHEMT Gilbert Cell Mixer for Intelligent Transportation System Radar Receiver. Master’s Thesis.

[12] Arun J., Ezra K., Nithin M., Ravi S. (2013). Design and Analysis of Double Balanced Gilbert Cell CMOS Mixer for Heterodyne receivers. Int. J. Appl. Eng. Res..

[13] Hu X. (2017). RF CMOS Tunable Gilbert Mixer with Wide Tuning Frequency and Controllable Bandwidth: Design Sythesis and Verification. Master’s Thesis.

[14] Ellinger F., Rodoni L.C., Sialm G., Kromer C., von Buren G., Schmatz M.L., Menolfi C., Toifl T., Morf T., Kossel M. (2004). 30–40-GHz drain-pumped passive-mixer MMIC fabricated on VLSI SOI CMOS technology. IEEE Trans. Microw. Theory Technol..

[15] Yang H.-Y., Tsai J.-H., Huang T.-W., Wang H. (2012). Analysis of a new 33–58-GHz doubly balanced drain mixer in 90-nm CMOS technology. IEEE Trans. Microw. Theory Technol..

[16] Sedighi S., Hashemipour O., Dousti M. (2016). A 2.4-GHz highly linear derivative superposition Gilbert cell mixer. Turk. J. Electr. Eng. Comput. Sci..

[17] Murad S.A.Z., Shahimin M.M., Pokharel R.K., Kanaya H., Yoshida K. (2012). Linearity improvement of 5.2-GHz CMOS up-conversion mixer for wireless applications. Microw. Opt. Technol. Lett..

[18] Zhang F., Skafidas E., Shieh W., Yang B., Wicks B.N., Liu Z. A 60-GHz double-balanced mixer for direct up-conversion transmitter on 130-nm CMOS. Proceedings of the 2008 IEEE Compound Semiconductor Integrated Circuits Symposium.

[19] Won Y.S., Kim C.H., Lee S.G. (2015). A 24 GHz Highly Linear Up-Conversion Mixer in CMOS 0.13 *μ*m Technology. IEEE Microw. Wirel. Components Lett..

[20] Wan Q., Wang C., Sun J. (2013). Design of a low voltage highly linear 2.4 GHz up-conversion mixer in 0.18 *μ*m CMOS technology. Wirel. Pers. Commun..

[21] Siddique A., Delwar T.S., Ryu J.Y. (2021). A high-linearity high-gain up-conversion mixer for 24 GHz automotive radar applications. Electron. Lett..

[22] Xavier B.A., Sullivan P.J., Fransis B., Ku W. A 0.9 V 960 MHz CMOS radio front end employing a doubly balanced transconductance mixer. Proceedings of the Solid-State Circuits Conference.

[23] Lin Y.S., Wen W.C., Wang C.C. (2013). 13.6 mW 79 GHz CMOS up-conversion mixer with 2.1 dB gain and 35.9 dB LO-RF isolation. IEEE Microw. Wirel. Components Lett..

[24] Chen A.Y.K., Baeyens Y., Chen Y.K., Lin J. (2011). An 80 GHz High Gain Double-Balanced Active Up-Conversion Mixer Using 0.18 *μ*m SiGe BiCMOS Technology. IEEE Microw. Wirel. Components Lett..

[25] Lee C.P., Behzad A., Ojo D., Kappes M., Au S., Pan M.A., Carter K., Tian S. A highly linear direct-conversion transmit mixer transconductance stage with local oscillation feedthrough and I/Q imbalance cancellation scheme. Proceedings of the 2006 IEEE International Solid State Circuits Conference—Digest of Technical Papers.

[26] Syu J.-S., Meng C. (2007). 2.4/5.7 GHz dual-band high linearity Gilbert upconverter utilizing bias-offset TCA and LC current combiner. IEEE Microw. Compon. Lett..

[27] Chiou H.-K., Chou H.-T. (2013). A 0.4 V microwatt power consumption current-reused up-conversion mixer. IEEE Microw. Compon. Lett..

[28] Codega N., Rossi P., Pirola A., Liscidini A., Castello R. (2014). A currentmode, low out-of-band noise LTE transmitter with a class-A/B power mixer. IEEE J. Solid-State Circuits.

[29] Li J., Gu Q.J. (2019). Harmonic-based nonlinearity factorization of switching behavior in up-conversion mixers. IEEE Trans. Circuits Syst. Reg. Pap..

[30] Wang X., Dengi A., Kiaei S. (2004). A high IIP3 -band BiCMOS mixer for radar applications. Proc. Int. Circuits Syst. Symp..

[31] Gilbert B. (1997). The micromixer: A highly linear variant of the Gilbert mixer using a bisymmetric class-AB input stage. IEEE Solid-State Circuits.

[32] Tseng S.C., Meng C.C., Chang C.-H., Wu C.-K., Huang G.-W. (2006). Monolithic broadband Gilbert micromixer with an integrated Marchand balun using standard silicon ic process. IEEE Trans. Microw. Theory Technol..

[33] Sivonen P., Vilander A., Parssinen A. (2005). Cancellation of second orderintermodulation distortion and enhancement of IIP2 in common source and common-emitter RF transconductors. IEEE Trans. Circuits Syst. Reg. Pap..

[34] Bao M., Li Y., Cathelin A. A 23 GHz active mixer with integrated diode linearizer in SiGe BiCMOS technology. Proceedings of the 33rd European Microwave Conference Proceedings (IEEE Cat. No.03EX723C).

[35] Tseng S.-C., Meng C.C., Wu C.-K. (2008). GaInP/GaAs HBT wideband transformer Gilbert downconverter with low voltage supply. Electron. Lett..

[36] Chen J.H., Kuo C.C., Hsin Y.M., Wang H. A 15–50 GHz broadband resistive FET ring mixer using 0.18 *μ*m CMOS technology. Proceedings of the 2010 IEEE MTT-S International Microwave Symposium.

[37] Lai I.C., Fujishima M. An integrated 20–26 GHz CMOS up-conversion mixer with low power consumption. Proceedings of the 32nd European Solid-State Circuits Conference.

[38] Comeau J.P., Cressler J.D. (2006). A 28-GHz SiGe up-conversion mixer using a series-connected triplet for higher dynamic range and improved IF port return loss. IEEE J. Solid-State Circuits.

[39] Lin Y.H., Li Y.C., Lin W.J., Tsai J.H., Alshehri A., Almalki M., Sayed A., Huang T.W. A Ka-band High Linearity Up-Conversion Mixer with LO Boosting Linearization Technique. Proceedings of the 2018 48th European Microwave Conference (EuMC).

[40] Madihian M., Desclos L., Maruhashi K., Onda K., Kuzuhara M. (1995). A monolithic AlGaAs/InGaAs upconverter IC for K-Band wireless networks. IEEE Trans. Microw. Theory Tech..

[41] Tsai J.-H., Lin W.-H., Huang C.-J. Design of 15–34 GHz low-power up-conversion ring mixer using 0.18 *μ*m CMOS technology. Proceedings of the 2016 IEEE International Symposium on Radio-Frequency Integration Technology (RFIT).

[42] Aparin V., Larson L.E. (2005). Modified derivative superposition method for linearizing FET low-noise amplifiers. IEEE Trans. Microw. Theory Tech..

[43] Chen Z., Liu Z., Jiang Z., Liu P., Liu H., Wu Y., Zhao C., Kang K. A 27.5–43.5 GHz high linearity up-conversion CMOS mixer for 5G communication. Proceedings of the 2017 IEEE Electrical Design of Advanced Packaging and Systems Symposium (EDAPS).

[44] Valdes-Garcia A., Reynolds S., Plouchart J.-O. 60 GHz transmitter circuits in 65 nm CMOS. Proceedings of the 2008 IEEE Radio Frequency Integrated Circuits Symposium.

[45] Verma A., O K.K., Lin J. (2006). A low-power up conversion CMOS mixer for 22–29-GHz ultra-wideband applications. IEEE Trans. Microw. Theory Tech..

[46] Zhu F., Wang K., Wu K. (2019). A reconfigurable low-voltage and low-power millimeter-wave dual-band mixer in 65-nm CMOS. IEEE Access.

[47] Motieifar A., Pour Z.A., Bridges G., Shafai C., Shafai L. An ultra wideband mixer with integrated impedance-matching circuit. Proceedings of the 2006 12th International Symposium on Antenna Technology and Applied Electromagnetics and Canadian Radio Sciences Conference.

[48] Wan Q., Wang C., Yu F. (2012). Design of a 2.4 GHz High-Performance Up-Conversion Mixer with Current Mirror Topology. Radioengineering.

[49] Sapone G., Palmisano G. (2007). A 1.5-V 0.25-*μ*m CMOS up-converter for 3–5 GHz low-power WPANs. Microw. Opt. Technol. Lett..

